# Supplementary material for: Intact tactile anisotropy despite altered hand perception in complex regional pain syndrome: rethinking the role of the primary sensory cortex in tactile and perceptual dysfunction
Source: PeerJ. 2021 May 3;9:e11156. doi: 10.7717/peerj.11156 (PMC8101475; doi:10.7717/peerj.11156)
Supplement: Supplemental Information 1 [file peerj-09-11156-s001.docx]

**Supplementary section**

This section contains supplementary material on the depictive body representation measure (the visual scaling task) and the tactile distance judgement task.

**Body perception measured via the visual scaling task**

Figure 1 shows the photographs of the male hand that were used to assess hand perception (the visual scaling task). It shows hands in four different size manipulations. All participants were shown the hand images in randomized order and asked to pick which image best matched their perception of their own hand.


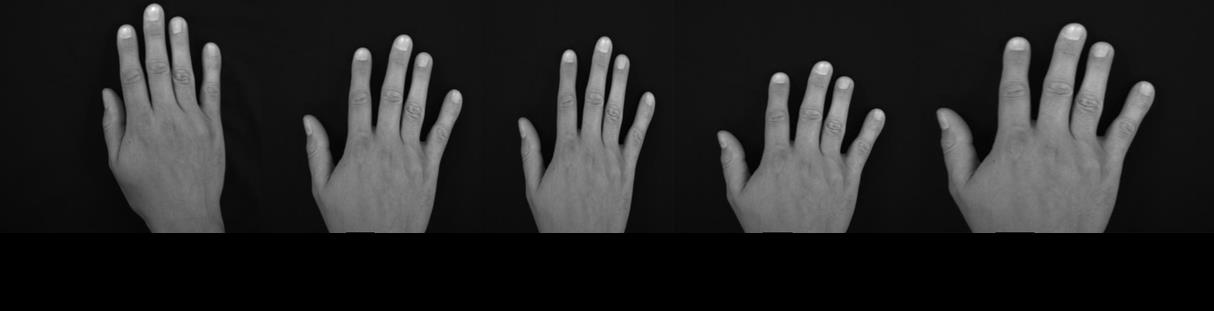


Figure 1: Exemplary of stimuli to assess and perception in the visual scale task. A template of male hand (here left), was presented in regular size (100%) and in four different size manipulations, either demagnified or magnified.

**Primary analysis (tactile distance judgements)**

The following three tables contain the general predictor variable set and odds for each variable including their beta/covariate values, 95% CI about the estimate and p-value for each model (Tables 1-3).

Table 1

*Anisotropic perception* bias (Primary outcome one) (perceiving across - distances as longer despite equal stimulus pair length)

|  | Univariate model | | | | | Multivariable model | | | |
| --- | --- | --- | --- | --- | --- | --- | --- | --- | --- |
| Variable | $e^{\hat{\beta}}$ | | 95% CI ($e^{\hat{\beta}}$) | Sig. | | $e^{\hat{\beta}}$ | 95% CI ($e^{\hat{\beta}}$) | | Sig. |
| **Anisotropic perception bias in all three groups** | | | | | | | | | |
| Constant |  | |  | |  | 4.702 | | 2.049 -10.795 | 0.000 |
| Participant Group |  | |  | |  |  | |  |  |
| 1 CRPS | 0.920 | | 0.479-1.768 | | 0.803 | 0.501 | | 0.215-1.171 | 0.111 |
| 2 Pain of other origin | 0.836 | | 0.502-1.393 | | 0.493 | 0.531 | | 0.244-1.159 | 0.112 |
| 3 Healthy | 1 | |  | |  |  | |  |  |
| Affectedness of the hand (affected or non-affected; c.q. dominant or non-dominant hand) | 0.789 | | 0.529-1.178 | | 0.247 | 0.783 | | 0.518-1.182 | 0.244 |
| Pain current | 1.017 | | 1.003-1.030 | | 0.013 | 1.012 | | 0.997-1.026 | 0.110 |
| Age | 0.996 | | 0.980-1.011 | | 0.569 | 1.008 | | 0.993-0.978 | 0.372 |
| Gender | 0.489 | | 0.305-0.782 | | 0.003 | 0,507 | | 0.300-0.857 | 0.011 |
| Pain average | 1.011 | | 0.998-1.024 | | 0.102 |  | |  |  |
| **Anisotropic perception bias in chronic pain** | | | | | | | | | |
| Constant |  |  | |  | | 1.433 | | 0.844 – 2.432 | 0.183 |
| Participant Group |  |  | |  | |  | |  |  |
| 1 CRPS | 1.100 | 0.625 - 1.938 | | 0.741 | | 0.813 | | 0.473-1.397 | 0.454 |
| 2 pain of other origin | 1 |  | |  | |  | |  |  |
| Affectedness of the hand (affected or non-affected; c.q. dominant or non-dominant hand) | 0.789 | 0.529-1.178 | | 0.247 | |  | |  |  |
| Gender | 0.462 | 0.282 – 0.754 | | 0.002 | | 0.609 | | 0.376 – 0.986 | 0.044 |
| Sign dynamic (touch) allodynia | 0.375 | 0.137- 1.026 | | 0.056 | | 0.609 | | 0.376-0.986 | 0.170 |
| Pain current | 1.017 | 1.003-1.030 | | 0.2013 | | 1.012 | | 1.000-1.023 | 0.054 |
| Pain average | 1.011 | 0.0998-1.024 | | 0.102 | |  | |  |  |
| Age | 0.993 | 0.978 - 1.008 | | 0.340 | |  | |  |  |
| Illness Duration | 1.001 | 0.996-1.005 | | 0.744 | |  | |  |  |
| DASH | 1.015 | 1.001-1.029 | | 0.036 | |  | |  |  |
| Body distortion FLF | 1.049 | 0.765-1.438 | | 0.765 | |  | |  |  |
| Body distortion BPS | 1.012 | 0.973-1.052 | | 0.543 | |  | |  |  |
| Sign pressure sensitivity | 1.372 | 0.643 - 2.929 | | 0.414 | |  | |  |  |

Table 2

*Predictors of tactile anisotropy (Primary outcome two to perceive across - stimuli as longer):*

|  | Univariate model | | | | | Multivariable model | | | |
| --- | --- | --- | --- | --- | --- | --- | --- | --- | --- |
| Variable | $e^{\hat{\beta}}$ | | 95% CI ($e^{\hat{\beta}}$) | Sig. | | $e^{\hat{\beta}}$ | 95% CI ($e^{\hat{\beta}}$) | | Sig. |
| **Tactile anisotropy in all groups** | | | | | | | | | |
| Constant |  | |  | |  | 1.972 | | 1.489-2.612 | 0.000 |
| Participant Group |  | |  | |  |  | |  |  |
| 1 CRPS | 1.035 | | 0.796-1.346 | | 0.797 | 0.623 | | 0.362-1.074 | 0.088 |
| 2 Pain of other origin | 0.941 | | 0.741-1.196 | | 0.621 | 0.632 | | 0.407-0.981 | 0.041 |
| 3 Healthy | 1 | |  | |  |  | |  |  |
| Affectedness of the hand (affected or non-affected; c.q. dominant or non-dominant hand) | 1.040 | | 0.883-1.226 | | 0.637 |  | |  |  |
| Gender | 0.782 | | 0.644-0.949 | | 0.016 | 0.688 | | 0.510-0.929 | 0.015 |
| Length difference | 2.478 | | 2.138-2.872 | | 0.000 | 2.496 | | 2.149-2.898 | 0.000 |
| Pain average | 1.007 | | 1.001-1.014 | | 0.030 | 1.010 | | 0.993-1.028 | 0.251 |
| Pain current | 1.007 | | 1.001-1.013 | | 0.031 | 0.998 | | 0.981-1.015 | 0.804 |
| Age | 0.999 | | 0.993-1.005 | | 0.724 |  | |  |  |
| **Tactile anisotropy in chronic pain** | | | | | | | | | |
| Constant |  |  | |  | | 1.178 | | 0.491-2.824 | 0.714 |
| Participant Group |  |  | |  | |  | |  |  |
| 1 CRPS | 1.100 | 0.842 - 1.436 | | 0.486 | | 1.053 | | 0.733-1.514 | 0.779 |
| 2 pain of other origin | 1 |  | |  | |  | |  |  |
| Affectedness of the hand (affected or non-affected; c.q. dominant or non-dominant hand) | 0.942 | 0.736-1.207 | | 0.637 | |  | |  |  |
| Pain average | 1.007 | 1.001-1.014 | | 0.030 | | 1.009 | | 1.000-1.018 | 0.063 |
| Illness Duration | 1.002 | 1.002-1.004 | | 0.125 | | 1.002 | | 0.999-1.005 | 0.231 |
| Length difference | 1.225 | 1.117 - 1.343 | | 0.000 | | 2.396 | | 2.011-2.856 | 0.000 |
| Pain current | 1.028 | 0.809-1.307 | | 0.819 | |  | |  |  |
| DASH | 1.007 | 0.998-1.015 | | 0.113 | |  | |  |  |
| Gender | 0.697 | 0.569-0.8533 | | 0.000 | |  | |  |  |
| Body distortion FLF | 1.083 | 0.9378-1.250 | | 0.279 | |  | |  |  |
| Body distortion BPS | 1.100 | 0.809-1.495 | | 0.544 | |  | |  |  |
| Sign dynamic (touch) allodynia | 1.318 | 0.884-1.964 | | 0.176 | |  | |  |  |
| Sign pressure sensitivity | 1.127 | 0.827-1.536 | | 0.0449 | |  | |  |  |

**Secondary outcome (tactile distance judgements)** Summary: These analyses show that the ability to accurately recognise and discern tactile stimuli is preserved in people with CRPS and PoP and comparable to that of pain-free healthy controls.. The rate of correctly recognised events was above common handled ranges of 75% showing accurate and preserved tactile sensitivity discrimination in all participants.

Secondary outcome one Across the sample, participants correctly identified 78% (95% CI: 76.2-79.9%) of the longer stimuli on the affected (or dominant) hand and 78.5% (95% CI: 76.7-80.3%) of the longer stimuli on the non-affected hand, when length difference between stimulus pairs (Figure 1) was truly discrepant (see Table 2). Among all groups, the accuracy of distance perception independent of orientation did not differ between the affected (dominant) hand and the non-affected (non-dominant) hand (χ2 (1): 0.754, p=0.784). Bilateral tactile accuracy (correctly detecting a wider stimulus pair when the stimulus pair is farther apart) increased as a function of length difference (OR: 1.235; CI: 1.144-1.334; p=0.000, see Table 1 of the supplementary section) in all groups.

Patient specific-clinical variable set

The best fit of data (QUICC: 2483.94) was approached by a model that includes affectedness of hand, presence of pain, illness duration, age and length difference. Again, bilateral tactile accuracy increased with increment of length discrepancy (OR: 1.228, 95%CI: 1.121-1.346, p=0.000). Importantly, clinical signs and symptoms (i.e. illness duration, pressure sensitivity, allodynia, motor impairment, distorted hand perception) did not significantly increase the variance explained in accurate distance perception judgment (see Table 1 of supplementary section).

Table 3

*Secondary outcome one: predictors of accurate length perception (independent of orientation)*

|  | Univariate model | | | | | Multivariable model | | | |
| --- | --- | --- | --- | --- | --- | --- | --- | --- | --- |
| Variable | $e^{\hat{\beta}}$ | | 95% CI ($e^{\hat{\beta}}$) | Sig. | | $e^{\hat{\beta}}$ | 95% CI ($e^{\hat{\beta}}$) | | Sig. |
| **Accuracy of tactile length perception in all groups** | | | | | | | | | |
| Constant |  | |  | |  | 5.392 | | 2.897-10.036 | 0.000 |
| Participant Group |  | |  | |  |  | |  |  |
| 1 CRPS | 0.689 | | 0.421 - 1.126 | | 0.137 | 0.612 | | 0.272-1.379 | 0.236 |
| 2 pain of other origin | 0.806 | | 0.492 – 1.320 | | 0.391 | 0.861 | | 0.446-1.662 | 0.655 |
| 3 Healthy | 1 | |  | |  |  | |  |  |
| Age | 0.992 | | 0.979 - 1.006 | | 0.273 | 0.994 | | 0.981-1.008 | 0.413 |
| Pain current | 1.007 | | 0.995 - 1.019 | | 0.244 | 1.003 | | 0.990-1.017 | 0.633 |
| Length difference | 1.235 | | 1.143-1.334 | | 0.000 | 1.235 | | 1.144-1.334 | 0.000 |
| Pain average | 1.006 | | 0.994 - 1.019 | | 0.303 |  | |  |  |
| Affectedness of the hand (affected or non-affected; c.q. dominant or non-dominant hand) | 1.025 | | 0.800 - 1.312 | | 0.846 |  | |  |  |
| Gender | 1.062 | | 0.638-1.766 | | 0.818 |  | |  |  |
| **Accuracy of tactile length perception both patient groups** | | | | | | | | | |
| Constant |  |  | |  | | 6.92 | | 2.32-20.622 | 0.001 |
| Participant Group |  |  | |  | |  | |  |  |
| 1 CRPS | 1.411 | 0.680 - 2.927 | | 0.356 | | 0.595 | | 0.321-1.103 | 0.099 |
| 2 pain of other origin | 1 |  | |  | |  | |  |  |
| Age | 1.165 | 0.841-1.614 | | 0.358 | | 0.991 | | 0.976-1.006 | 0.232 |
| Gender | 1.197 | 0.973-1.003 | | 0.118 | |  | |  |  |
| Pain current | 1.007 | 0.995-1.019 | | 0.244 | | 1.003 | | 0.990-1.016 | 0.675 |
| Pain average | 1.006 | 0.994-1.019 | | 0.303 | |  | |  |  |
| Affectedness of the hand (affected or non-affected; c.q. dominant or non-dominant hand) | 1.165 | 0.841 – 1.614 | | 0.358 | | 1.170 | | 0.839-1.630 | 0.355 |
| Illness Duration | 0.997 | 0.993-1.001 | | 0.154 | | 0.997 | | 0.993-1.001 | 0.137 |
| Length difference | 1.225 | 1.117 - 1.343 | | 0.000 | | 1.228 | | 1.120-1.346 | 0.000 |
| DASH | 1.004 | 0.998-1.019 | | 0.641 | |  | |  |  |
| Body distortion FLF | 1.075 | 0.752-1.537 | | 0.693 | |  | |  |  |
| Body distortion BPS | 1.013 | 0.973-1.054 | | 0.525 | |  | |  |  |
| Sign dynamic (touch) allodynia | 0.965 | 0.532-1.657 | | 0.898 | |  | |  |  |
| Sign pressure sensitivity | 1.372 | 0.643 - 2.929 | | 0.414 | |  | |  |  |

Secondary outcome two: Accuracy of length discrimination in the across-orientation

The proportion of across-stimulus pairs that were correctly identified as longer than the along-stimulus pairs did not differ between the groups on both hands (χ2 (1): 0.254, p=0.614, Table 2). As expected, correctly detecting a farther apart distanced stimulus pair in the across-orientation (when the across-orientation stimulus pair is farther apart) increases with increasing length difference (OR: 2.44 CI 95%: 2.117-2.821; p=0.000) on both hands in all groups.

Patient specific-clinical variable set

Here, the best model of fit (QUICC: 2386.763) comprised gender (OR: 0.792; 0.589-1.065; p=0.122), illness duration (OR: 1.003; CI 95%: 1.000-1.006; p=0.059) and subjective perception of disability (DASH; OR: 1.006; CI 95%: 0.993-1.018; p= 0.361). Importantly, neither clinical nor sensory signs, nor distorted body perception, influence accuracy of tactile anisotropy.
